# Improvements and Inventions

**Published:** 1855-12

**Authors:** 


### IMPROVEMENTS AND INVENTIONS.

### VENTILATION.—DUVOIR'S SYSTEM.

A new system of ventilation has recently been invented, and extensively adopted in Paris. M. Duvoir is the inventor.

The peculiarity of this system is, that it supplies two processes at one and the same time; namely, warming and ventilation.

The plan is simple. At one end of the building that is to be warmed and ventilated by this system, there is sunk in the soil a chamber containing a grate, on which is placed a bell-shaped double walled iron vessel, filled with water. From this reservoir, and running upwards in the chimney of the furnace, are three pipes which are made spiral, so as to offer a larger surface for obtaining heat from the smoke ascending around them.

The spiral pipes convey from the lower reservoir an upward current of heated water, to an open reservoir at the top and centre of the building. From thence the water is distributed downwards, and in its heated state, by other pipes, into every part of the building. In each room is a kind of water stove, through which the water plays, and from which sufficient heat is radiated to warm the apartment. After the water from the upper reservoir is fully distributed over the building, it is collected by a common pipe, and is conveyed into the lower reservoir over the furnace to ascend and circulate, give up its caloric in some part, and return for more.

As the water in its journey is only partially cooled, the amount of fuel required in the furnace is moderate. By this simple means the warming process is carried on; but more than this is effected, for ventilation is at the same time secured.

The open reservoir at the top of the building, which, as we before said, was kept filled with hot water, is surrounded by a chamber, out of which a shaft rises. This chamber receives the air from all the rooms of the building, by means of a series of ventilating tubes or shafts, one of which runs from each room upwards to the top of the building, where it enters at a right angle into a common transverse shaft communicating with the chamber.

From the extensive radiation of heat from the open reservoir, the air in the chamber is expanded, and a constant current from the building below upwards through the shaft is sustained.

Each of the ventilating shafts in the separate rooms is provided with a square opening at the bottom and the top. These admit of being closed at pleasure. In the winter, the upper one is closed, and the air therefore with which the room is charged from without has to find its escape by a downward movement to the opening at the bottom of the room, and so upwards through the shaft. In the summer, the lower opening is closed, and the upper one is opened, so that the current is directed upwards into the ascending shaft.

The advantages which are said to arise from this system are: 1st, that it ensures free ventilation; 2nd, that it warms and ventilates at the same time; 3rd, that it is cleanly and inexpensive; 4th, that in hospital wards, where the emanations from the sick are offensive or pernicious, such emanations can be borne away directly from above downwards, by having the upper opening in the ventilating shafts in each ward closed, and the lower one open. The wards are thus constantly swept clean of all hurtful gaseous products.

The mode of ventilation here described has been applied in Paris to many public buildings with entire success. There lies before us at this time a diagram showing this principle as applied to the "Hôpital de Lariboisière", and also several reports from distinguished judges of the value of its action and of its successful application. These are all candid, and, at the same time, are in general decidedly favourable.

We hope, ere long, to see M. Duvoir's principle introduced into this country. If its success were confirmed, it would prove a valuable acquisition, and not an unprofitable one in a business point of view.

For the intimation of this mode of ventilation, for the inspection of the diagram above referred to, and for the perusal of the documents descriptive of the plan, we are deeply indebted to Dr. Waller Lewis, whose knowledge of the sanitary state of the capital of France, and of the various improvements in sanitation, is of a high order. Dr. Lewis, after having seen Duvoir's system in operation, expresses himself to us as perfectly satisfied in regard to its utility.

---

### A NEW MODE OF VENTILATION.

BY C. COWAN, M.D.

In the June number of the Journal of Public Health, there is an article on the "Air-Syphon Ventilator," giving an account of a mode of ventilation originated by Dr. Chowne, and founded upon the singular fact, that if a tube of the syphon shape be placed in a room with its long end uppermost, a current of air will immediately play through it in the downward direction of the short, and in the upward direction of the long leg of the tube. Several useful suggestions are also made, which, from their simplicity and cheapness, are well worthy of trial.

The object of the present communication is to direct the attention of practical men to another, and, if possible, still more simple process, by which, under various circumstances, ventilation may be effectually promoted; viz., by simply *bisecting all tubes or outlets through which a current of air is desirable.*

Let any experimenter take an ordinary lamp chimney-glass, and hold it over some paper, in that state of ignition most adapted for the production of smoke, leaving a small space for the entrance of air at the bottom; all the movements within the tube will, of course, become visible to the eye. Under strong heat or great upward draught no material obstacle to the exit of the smoke will be observed; but, under other conditions, its passage upward will be slow and uncertain, with sometimes an escape from beneath.

A rotatory movement, with a variety of secondary disturbances, will be perceived, the result of the collision of opposing currents.

In this stage of the process, let a piece of writing-paper, adapted to the length and diameter of the tube, be passed down the centre; and immediately order replaces disorder. Instead of conflicting eddies, the smoke at once ascends in a steady and rapid stream, on *one* or other side of the septum; a downward current, more or less active, being established on the other. One half of the tube is thus perfectly free from smoke; and, should the respective currents change sides, only momentary confusion results.

The above experiment was prompted by the statement of Commander Priest, a most intelligent officer of the royal navy, who found that by straining a piece of canvass vertically across the deck, opening to the engine room, the temperature below was rapidly lowered. Of this the men were so conscious, that they never failed to avail themselves of so simple a contrivance. Why such a result should follow, is clear from the experiment we have now detailed; and the application of the principle I believe to be both multiform and valuable.

Smoky chimneys, for example, with their legionary train of evils and inconveniences, would be impossible, were their spaces properly subdivided; for no disproportion in the relative strength of either upward or downward currents would prevent their independent establishment. The short and ever smoking chimneys of small tenements and upper chambers might thus be made efficient; and in cases where bisecting the tube was impracticable, suspending a central tube would probably succeed. The pipings of stoves, if so constructed, would be far more certain in their action, while the downward draught might be easily converted into an efficient bellows for the fire.

All openings, tubular or other, might be thus rendered self-adaptive; and orifices intended for the escape, could be converted into channels for the admission of air. Gas-lights, with properly-adjusted tubes, would do the work of ventilators, instead of, as at present, acting most injuriously to health and life. In the cabins and holds of vessels, important advantages might be secured; but when once the difference in the mode of acting of a single and a divided space is clearly apprehended, the question how far it is applicable to special conditions can be easily decided.

The above suggestions are not advanced with the slightest claim to originality. They may, probably, have presented themselves to the minds of many; but, having never met with their practical enforcement, and having confidence in their truth and importance, I forward them as not unworthy of insertion in, or unadapted to the spirit of this excellent periodical.

Reading, July 1855.

[The facts described by our friend Dr. Cowan have, as he supposes, presented themselves to other minds. Indeed, the principle laid down by Dr. Cowan has been adapted to ventilation by Mr. Watson of Halifax, under the title of the Diaphragm system of ventilation, or some similar term. In company with Dr. Lewis, the Medical Officer of the Post-office, we had the advantage, some little time ago, of seeing Mr. Watson's plan in operation, he having had the opportunity of fitting it up in one of the lower vaults, or kitchens, of the General Post-office, which at the present time are in a deplorable state of ventilation. Whether from some accidental cause or fault in construction, we cannot say, but certain it is, that, from all we could learn, Mr. Watson's plan did not work so satisfactorily as might have been expected. It seemed to permit of strong downward currents, and we could not distinguish, by a piece of lighted paper, any difference of draught upwards on either side of the diaphragm or dividing partition. We would not, however, speak slightingly of this system without further evidence of a practical kind.—Ed.]

---

